# Evidence of Infectious Asthma Phenotype: *Chlamydia*-Induced Allergy and Pathogen-Specific IgE in a Neonatal Mouse Model

**DOI:** 10.1371/journal.pone.0083453

**Published:** 2013-12-20

**Authors:** Katir K. Patel, Wilmore C. Webley

**Affiliations:** Department of Microbiology, University of Massachusetts, Amherst, Massachusetts, United States of America; Alabama State University, United States of America

## Abstract

Asthma is a chronic respiratory disease whose etiology is poorly understood. Recent studies suggest that early-life respiratory infections with atypical bacteria may play an important role in the induction or exacerbation of chronic respiratory disease. The current study utilized a neonatal mouse ovalbumin (OVA) sensitization model of asthma to determine the course of early-life respiratory tract infection by *Chlamydia*. Neonatal (day 1) and adult (6 wks) BALB/c mice were infected intranasally with *Chlamydia (MoPn)* and 7 weeks later were sensitized and challenged with ovalbumin. Allergic airway disease was characterized by examination of serum and bronchoalveolar lavage fluid (BAL) cellularity, cytokine production and antibody response. The presence of *Chlamydia* was determined by PCR and culture. Ova-specific IgE was quantified by ELISA and *Chlamydia*-specific IgE was determined via Western blot analysis. Chlamydial infection in neonatal mice induced increased production of Th_2_ cytokines (IL-4, 5, 10, and 13) in both BAL and serum, while infected adult mice produced increased Th_1_ cytokines (IL-2, IFN-γ). The BAL from infected neonates contained significantly elevated levels of eosinophils compared to infected adult mice. Although adult mice cleared the infection ∼30 days post infection (pi), neonates were still infected 66 days after initial infection. *Chlamydia*-specific IgE was detected in both the BAL and serum of neonatal mice beginning 28 days post infection, however, infected adult mice did not produce *Chlamydia*-specific IgE antibodies over the course of the study. When allergic airway was induced using Ova, infected neonatal mice increased their production of IL-4, IL-5 and IL-13 by >2 fold compared to uninfected controls and infected adult groups. Our findings demonstrate that early-life *Chlamydia* infection induces a Th_2_-dominant cytokine response in the airways of neonatal mice, leading to chronic infection. More significantly, early life respiratory colonization with *Chlamydia* elicits pathogen-specific IgE production, which further supports an infectious asthma phenotype.

## Introduction

According to the most recent available data, the total incremental cost of asthma to society is approximately $56 billion, with productivity losses due to morbidity accounting for $3.8 billion and productivity losses due to mortality accounting for $2.1 billion [1]. Over the last 2 to 3 decades there has been a significant increase in asthma prevalence in Western countries and recent data suggest that while these levels might be peaking, many low and middle income countries are now beginning to experience increases in prevalence [2]. Until recently it was widely believed that asthma was an atopic disease caused by allergen exposure and that the global increases in asthma prevalence were due to increases in exposure to aeroallergens which lead to eosinophilic infiltration, mast cell degranulation, hyper-responsiveness and airflow obstruction; and was fundamentally linked to a patient’s genetic inheritance [2], [3]. However, it is becoming increasingly evident that this allergen-mediated, eosinophilic airways inflammation model is an overly simplified explanation of this very complex disease and that no single etiology can be defined to date [4]. While it is indisputable that there are many clear cases of allergen exposure leading to asthma development in adults, overall there is little evidence that allergen exposure is a major primary cause of asthma in children, and even some evidence that allergen exposure early in life may have a protective effect [3]. Moreover, recent studies support the conclusion that non-allergic or non-eosinophilic airways inflammation may account for over half of all asthma cases [5]. Eosinophilic asthma is now classified as a distinct asthma phenotype that is characterized pathologically by significant basement membrane thickening and pharmacologically by corticosteroid responsiveness. In contrast, non-eosinophilic asthma, that includes most patients with severe disease, has very little basement membrane thickening and appears to be relatively corticosteroid resistant [6]. Published reports strongly suggest that despite clinically similar features, not all asthma are the same and patients may therefore benefit from personalized treatment. Moreover, surveys have consistently shown that many patients with asthma do not have their disease well controlled. A recent CHOICE survey study concluded that of all asthma patients on controllers, only 14.3% were well controlled [7]. However, before these patients can be effectively treated, a better understanding of non-allergic asthma etiology is necessary.

Since allergic asthma seems to be a Th2-disease, immunomodulating factors such as early childhood infections, LPS-exposure or other factors influencing gene-environment interaction and individual susceptibility might be relevant for the development of childhood asthma [8]. The hygiene hypothesis suggests that early-life infections are crucial in shaping and developing dominant immune responses; it also suggests that exposure to Th1-inducing pathogens is essential so that neonates can mount protective Th1 responses later in life [9]. It now appears that the timing of exposure to infection, the virulence properties of the infectious agent, and the genetic susceptibility of the host, all play an important role in the future development of allergic disease [9]. Although chlamydial infections induce and are ultimately cleared by Th1-mediated immune responses, clinical studies link chlamydial lung infection with the development of asthma in children[10], [11]. Indeed, a recent study from our lab showed that over 68% of children with asthma harbored viable *Chlamydia* in their lungs and that atopy was strongly associated with infection [12]. These data suggest that only in some predisposed individuals does infection induce Th2 responses [12]. To this end Hansbro et al have proposed two hypotheses to explain the association between Th1-inducing infections and asthma [13]. The first is that neonatal responses to infection are highly polarized towards Th2 immunity and thus early life chlamydial infection in neonates reinforces rather than suppresses this response, which leads to atypical Th2 responses to the infection[13]. This has the potential to drive the immune system to develop an allergic phenotype, which can ultimately lead to persistent infection, increasing the severity of Th2-type inflammatory responses to environmental antigens, and drive asthmatic disease. The second hypothesis is that Th1-inducing infections may cause a generalized inflammation of the airways that leads to the exacerbation of allergen-induced inflammation and asthma later in life [13]. These early life infections, according to Starkey et al [14], might promote permanent deleterious changes in immunity, lung structure and function that predispose to, or increase the severity of chronic respiratory diseases later in life. It is likely that both of these might be playing a role here.

Previously published data from our lab demonstrate strong evidence linking *Chlamydia pneumoniae* infection with the development and exacerbation of asthma in pediatric patients. *C. pneumoniae* has, therefore, been associated with both protective (Th1) as well as pro-asthmatic (Th2) immune responses. As a corollary, and in an attempt to better understand the mechanisms involved, we utilized a mouse model to investigate whether chlamydial infection in early life stimulates protective immunity or drives Th2 responses leading to the development and/or exacerbation of asthma later in life. We utilized a murine ovalbumin (Ova)-induced allergic airway disease (AAD) model that was previously developed and refined by Horvat, et al [15] to determine the impact of early life chlamydial infection on the subsequent development of AAD in adulthood.

## Methods

### Ethics Statement

This study was carried out in strict accordance with the recommendations described in the Guide for the Care and Use of Laboratory Animals of the National Institute of Health. The study received prior approval from the University of Massachusetts Amherst Institutional Animal Care and Use Committee (Protocol #28-05-01R). Mice were housed in microisolator cages and all neonates remained with their parent until weaned. Animals were kept under controlled conditions of humidity, temperature and light (12-hour light/12-hour dark cycles). Food and water were available *ad libitum*. Our animal care facilities are accredited by the American Association for Accreditation of Laboratory Animal Care. Animal health status was evaluated on a daily basis by investigators and an animal care technician as well as twice weekly by a staff veterinarian. All personnel received training and had up-to-date certifications in laboratory animal handling and care. All procedures on live animals were carried out under ketamine/xylazine anesthesia and euthanasia was carried out via sodium pentobarbital overdose by trained animal care personnel under the direct supervision of a member of our veterinary staff. All efforts were made to minimize animal suffering during procedures and at the time of euthanasia.

### Mouse Models

For neonatal experiments, pregnant BALB/c mice were purchased from Jackson Labs (Bar Harbor, ME) and used with the approval of the University of Massachusetts Amherst IACUC. Within 1 hour of birth (Day 0) mice were infected intranasally (IN) with *C. muridarum,* formerly known as the mouse pneumonitis biovar of *C. trachomatis* (approximately 200 inclusion-forming units (IFU) in 5µl sucrose phosphate-glutamate buffer, SPG).

For adult experiments, 5 week old virgin female BALB/c mice were utilized. The groups of adult mice were treated in a similar manner as the newborns. Adult mice were anaesthetized by isoflurane inhalation, held in the upright position and the inoculum, 200 IFU in sucrose phosphate glutamate buffer (SPG, 50ul final volume), was pipetted onto the nares until the whole inoculum was inhaled. Control mice for both groups received equivalent volumes of SPG via a similar route.

Seven weeks post infection/placebo treatment (Day 50) mice were sensitized to Ova by intra-peritoneal (IP) injection of 50µg Ova in 200µl 0.9% sterile saline. Twelve days after sensitization (day 62) mice were challenged IN with Ova (10µg, 50µl PBS, for 4 consecutive days). One day later (day 5 post Ova challenge), mice were euthanized by sodium pentobarbital overdose and features of acute airway disease (AAD) were characterized.

### Chlamydial Infection

All mice were weighed initially and then daily. The rate of weight gain/loss was calculated (g/d) over the entire course of the study as an indicator of health status. Chlamydial lung infection was initially assessed by PCR and organism numbers in the lungs, liver and spleen were determined by tissue culture. Briefly, lung tissues was cut and weighed followed by homogenization in sterile SPG. Homogenates were centrifuged and the supernatants used for culture and DNA isolation.

Chlamydial organisms were cultured utilizing 24-well plates with 12 mm coverslips seeded with a semi-confluent monolayer of mouse macrophages (J774A.1) for 36 hours. Following incubation, cells were fixed and immunostained using Pathfinder antibody (Bio-Rad, Hercules, CA) according to manufacturer’s instructions and inclusion forming unit counts (IFUs) were done.

Genomic DNA was isolated from BAL & lung tissue using the QIAMP DNA Blood mini kit (Qiagen) PCR was used to detect chlamydial DNA. Previously published *Ct* primers P1 and Omp2 were used to amplify a 1,130bp fragment [16].

### Bronchoalveolar lavage fluid Lavage (BAL) and Cellularity Analysis

Bronchoalveolar lavage fluid (BAL) was obtained by cannulation of the trachea and lavaging the airways with 2×1 ml sterile saline. BAL cell counts were performed with a cell counting chamber (hemocytometer, improved Neubauer) under phase contrast microscopy with results expressed as “number of cells per mm^3^”. BAL differential counts were performed using Wright stained cytospin preparations of BAL. At least 200 cells were counted per slide to obtain statistically significant counts.

### Serum and BAL Antibodies

Serum antibody titer to *Chlamydia* as well as Ova was evaluated from tail bleeds taken on a weekly basis and at time of euthanasia. *Chlamydia* antibody titers were evaluated by enzyme linked ImmunoSorbent assay (ELISA). The ELISA wells were coated with purified chlamydial EBs (300 ug/ml) and serial dilutions of each mouse serum added. Following the required washes, bound primary antibodies were detected by an AP-conjugated goat anti-mouse secondary antibody. Quantitative assessment of OVA-IgE in the BAL fluid and serum was conducted using OVA-specific mouse-IgE ELISA kit from BD Biosciences, according to the manufacturer’s instructions**.** *Chlamydia*-specific IgE antibodies were detected using Western blot assay as previously described [17], [18].

### Cytokine Production in BAL

Total cytokines secreted in the lung milieu was determined through analysis of BAL fluid. Concentrations of IL-2, IL-4, IL-5, IL-12, IL-13, and IFN-γ were determined using OptEIA Mouse ELISA kits (BD Biosciences) according to manufacturer’s instructions.

### Cytokine Production in WBCs from Lymph Nodes

Upon euthanasia, mediastinal lymph nodes were collected from each mouse, pooled in pairs and homogenized by forcing them through cell sieves. The cells were pelleted and resuspended in RBC lysing buffer, followed by another centrifuge step and resuspension in DMEM culture media containing 5% FBS. Cell numbers were determined by counting on a hemocytometer and approximately 2×10^6^ cells/ml were used to seed 24-well plates and were then stimulated with Ova, formalin killed *Chlamydia,* J774A.1 cell lysates, or PBS. Supernatant was removed at 12h intervals over a 72h time period and frozen until time of testing. These supernatants were evaluated for production of IL-2, IL-4, IL-5, IL-12, IL-13 and IFN-γ using the BD Bioscience kit described in the previous section.

### Statistics

Results are presented as mean± SEM from each test and control group of mice (approximately 4 animals per group). Analyses were performed using the SPSS Graduate Pack 14 for Windows or Microsoft Excel and significant associations or differences are based on two tailed tests with p values <0.05.

## Results

### Chlamydial Lung Infection and Associated Pathology

**Figure 1:**
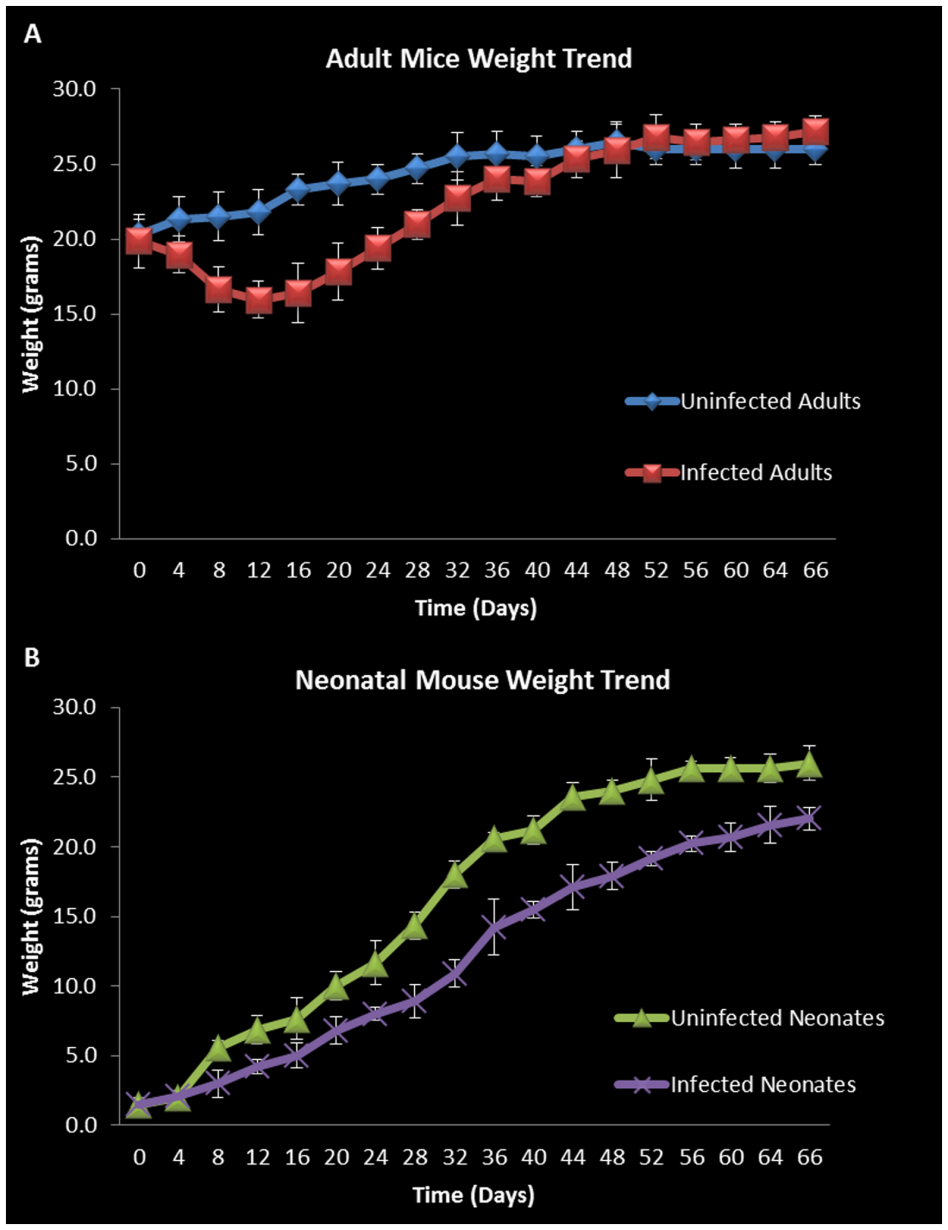
Adult and neonatal weight trends. Weight trend graphs show significant weight loss in *Chlamydia* infected adults vs. uninfected adults between days 8 and 28 post infection (on average 5.9 g smaller or 26% weight reduction). However, infected adults recovered lost weight by day 44 (A). Infected neonatal mice showed retarded weight gain throughout the course of the study compared to uninfected neonates but the most significant differences could be seen between days 32 and 52 (on average 6.1 g smaller (28%) (B). Each point on the curve is the average weight of animals in the specific group. Error bars represent standard error from the mean of at least 8 animals in each group at each time point.

The OVA sensitization model of asthma is widely used and well characterized displaying features very similar to those seen in human allergic asthma. We performed intranasal inoculation of the chlamydial organism or control in each of our mouse groups as described in the methods section and assessed the course of infection-related pathology as well as changes associated specifically with the induction of bronchial inflammation and airway hyperresponsiveness following Ova sensitization and challenge. Animals were weighted each day and the weight trend shows significant weight loss in *Chlamydia*-infected vs. uninfected adult mice starting at four days post infection. In adults the most significant weight loss was between days 8 and 28, where on average infected adults were 26% (5.9 g) smaller than their uninfected counter parts (Figure 1A). The most significant differences in weight gain were observed in neonates between days 32 and 52, where on average infected neonatal mice were 28% (6.1 g) smaller than uninfected neonates (Figure 1B). The rate of weight gain between infected and uninfected adult groups was not significantly different during the later parts of the time course (Figure 1A). Infected neonatal mice, however, demonstrated retarded weight gain throughout the course of the study compared to uninfected neonates (Figure 1B).

**Figure 2:**
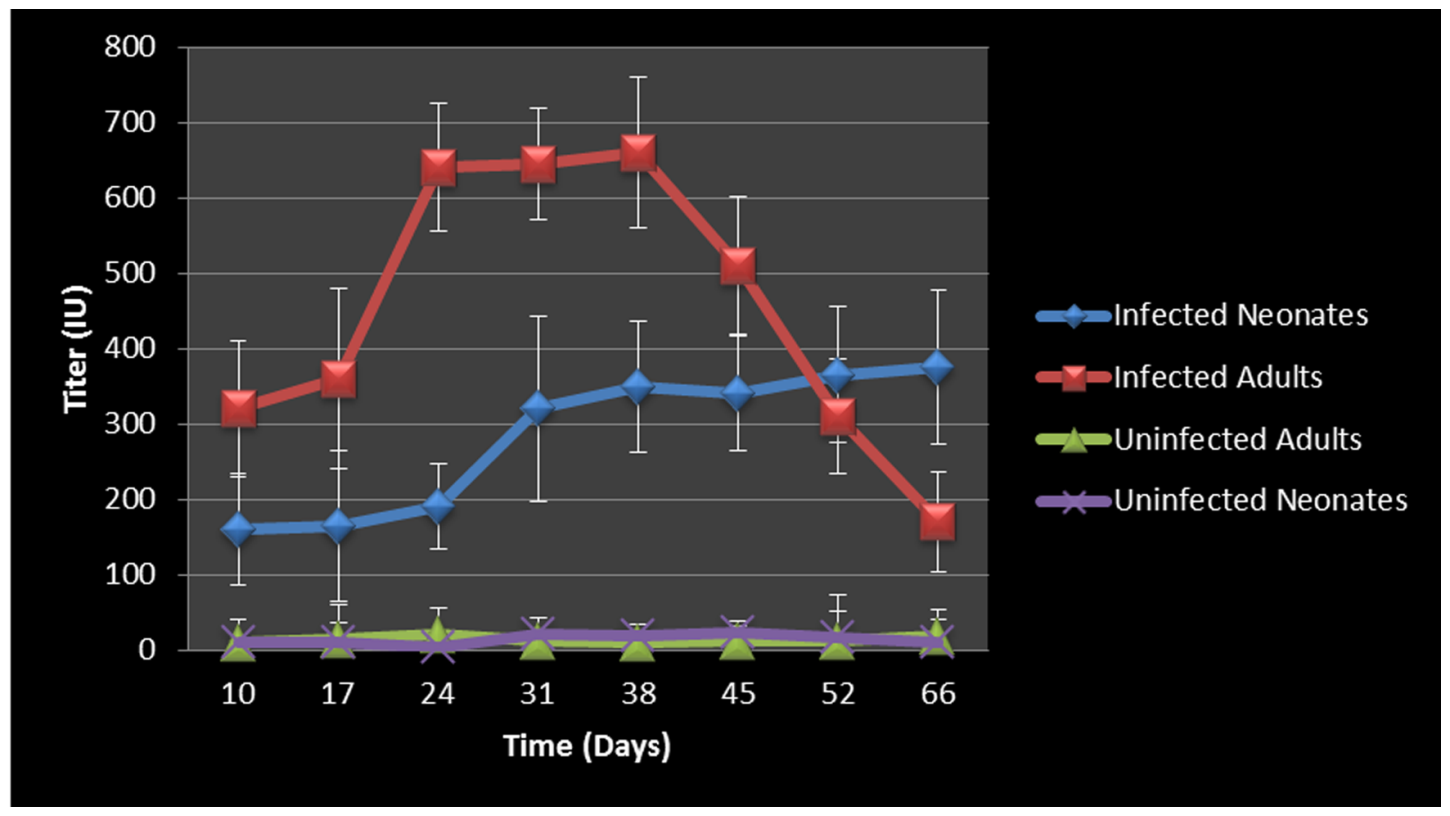
*Chlamydia* antibody titers over time. Antibody titer graph shows strong antibody responses to *Chlamydia* from infected adult mice but relatively decreased and/or retarded antibody titers in the infected neonatal mouse groups. Infected adult titers dropped once infections cleared, however infected neonatal mice did not clear the infection and their titers consistently increased over the course of the study. Each point on the graph represents the average serum titer from at least 8 animals, with sera run in duplicate. The error bars represent the standard error of the mean within each group at the specific time point.

In an effort to assess the humoral immune response to intranasal chlamydial challenge, we collected sera from each animal on a weekly basis and assayed for anti-*Chlamydia* antibody response. The data revealed strong antibody responses to *Chlamydia* infection in the adult mice. Neonatal animals whose immune system was not yet fully developed had an attenuated antibody response initially, but the response continued to increase for the duration of the study (Figure 2). Titers began to decrease in infected adult animals around the time of infection clearance; however infected neonatal mice did not clear the infection, explaining why the titers consistently increased over the course of the study.

**Figure 3:**
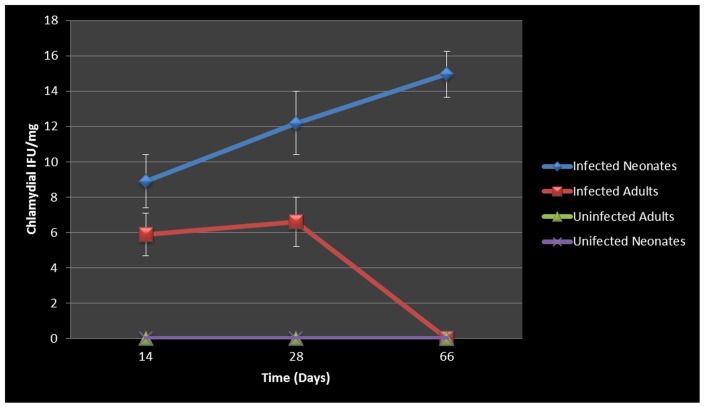
Cultured chlamydial carriage/concentration in the lungs. Infected adult mice were able to clear the chlamydial infection after day 28. Infected neonates were unable to clear the chlamydial infection during the course of this study and chlamydial concentration per/mg of tissue as well as total chlamydial carriage in lung continued increase over time.

The concentration of chlamydial organism in the lungs (IFU/mg) of neonatal animals increased significantly with time and there was almost a 2 fold increase from day 14 to day 66 (Figure 3). In contrast, infected adults displayed no significant increases in chlamydial IFU/mg during the course of the study, and ultimately cleared the infection 28 days pi. By day 7, chlamydial organisms could be found in the liver, spleen and peripheral blood (tail bleeds), demonstrating dissemination from the respiratory mucosa, leading to systemic infection.

### WBC and Cytokine Response to Respiratory Chlamydial Infection

**Figure 4:**
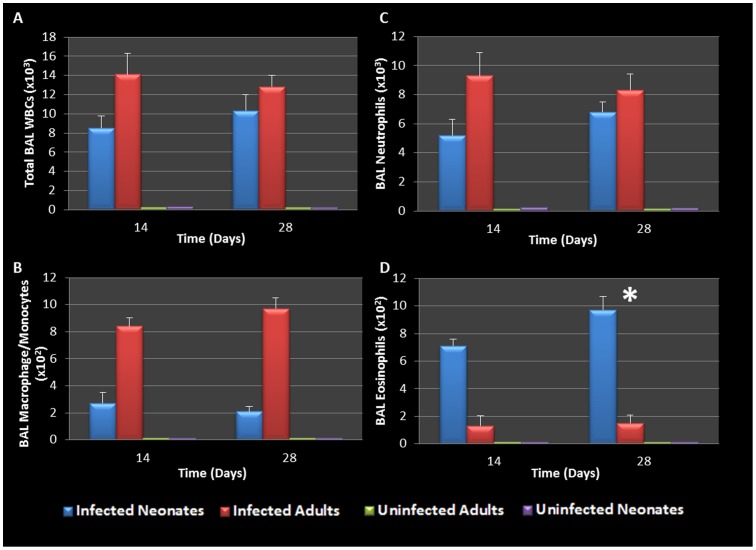
BAL cellularity during chlamydial infection. Elevated total WBCs and neutrophil levels were seen in infected neonatal and adult groups (A, C). Infected adults had elevated macrophage/monocytes (panel B), whereas infected neonates were characterized by significantly elevated eosinophil levels on day 28 pi compared to uninfected adults (P = 0.0449, panel D).

It is well established that genital *Chlamydia* infection induces a Th1 immune response characterized by IFN-γ secretion and is essential for clearance of this intracellular pathogen. It is also well documented that AAD is a Th2-driven disease characterized by the influx of Th2 cytokines, eosinophils and allergic airway hyperresponsiveness. We therefore sought to determine what would characterize lung infection with *Chlamydia*. The airway of *Chlamydia*-infected adults and neonatal mice was characterized by the accumulation of predominantly macrophages (9.7×10^2^) and neutrophils (8.3×10^3^) in the BAL fluid of adult mice 28 days post infection, along with markedly less eosinophils (1.5×10^2^). In contrast, at 28 days pi, infected neonatal mice presented with significantly elevated eosinophils (9.7×10^2^, P  =  0.0449) compared to infected adults. There was no significant difference in infected neonatal BAL neutrophils (6.8×10^3^), or macrophages (2.1×10^2^) compared to infected adults (Figure 4 A-D), however infected groups had significantly elevated levels of all cell types when compared to uninfected controls. This highlights a major difference in the early life response to chlamydial infection compared to adult counterparts.

**Figure 5:**
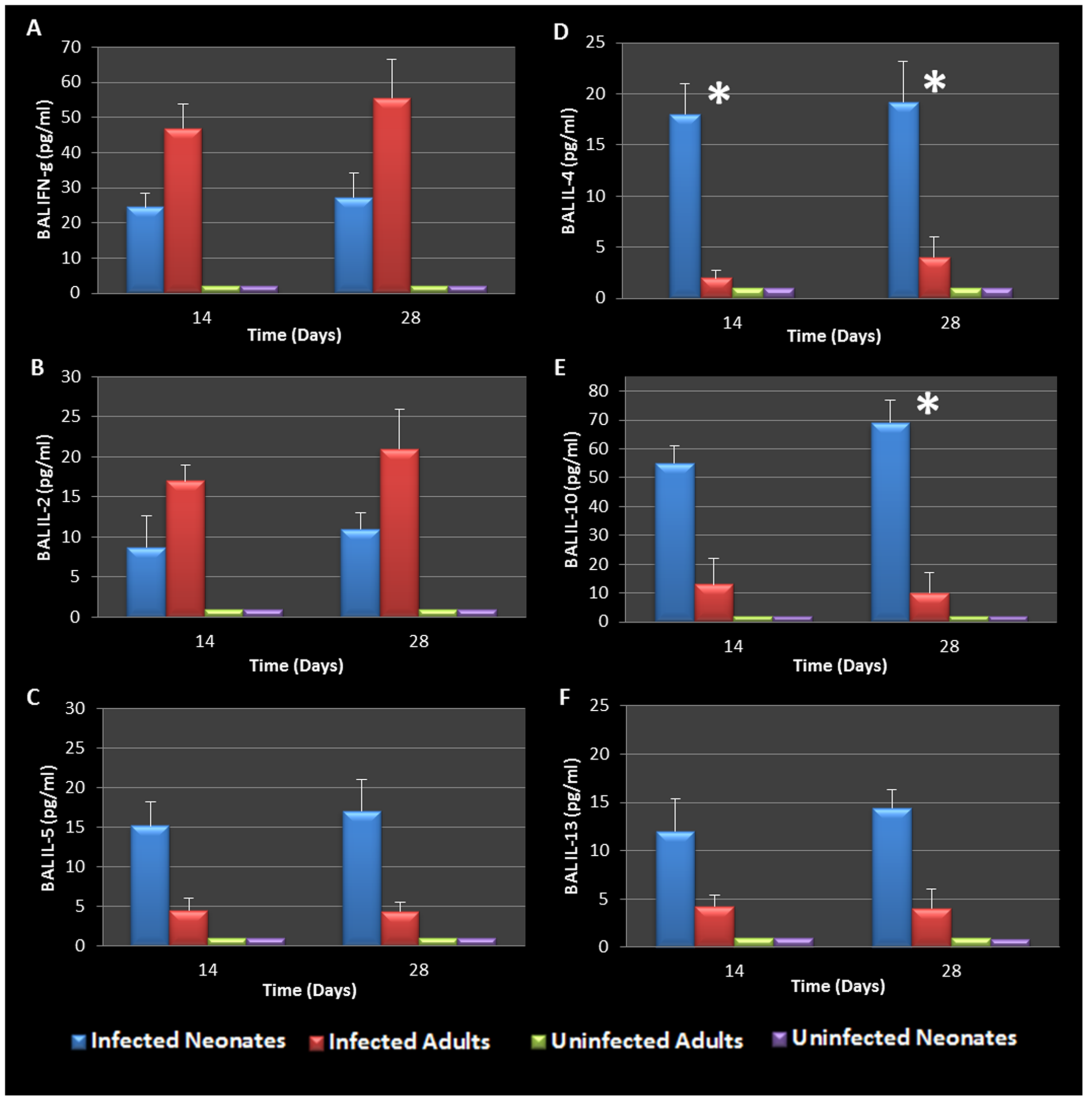
BAL cytokines during chlamydial infection. Infected adults displayed a more pronounced Th1 cytokine response (IFN-γ and IL-2) than infected neonates (A, B). Infected neonates responded to chlamydial infection with a robust Th2 cytokine response (IL-4, IL-10, IL-5, and IL-13) compared to their adult counterparts (C-F). Specifically, neonatal mice produced significantly elevated levels of IL-4 compared to infected adults 14 and 28 days post infection (P = 0.0312, 0.0248 respectively, D) as well as IL-10 28 days post infection (P = 0.0434 E).

**Figure 6:**
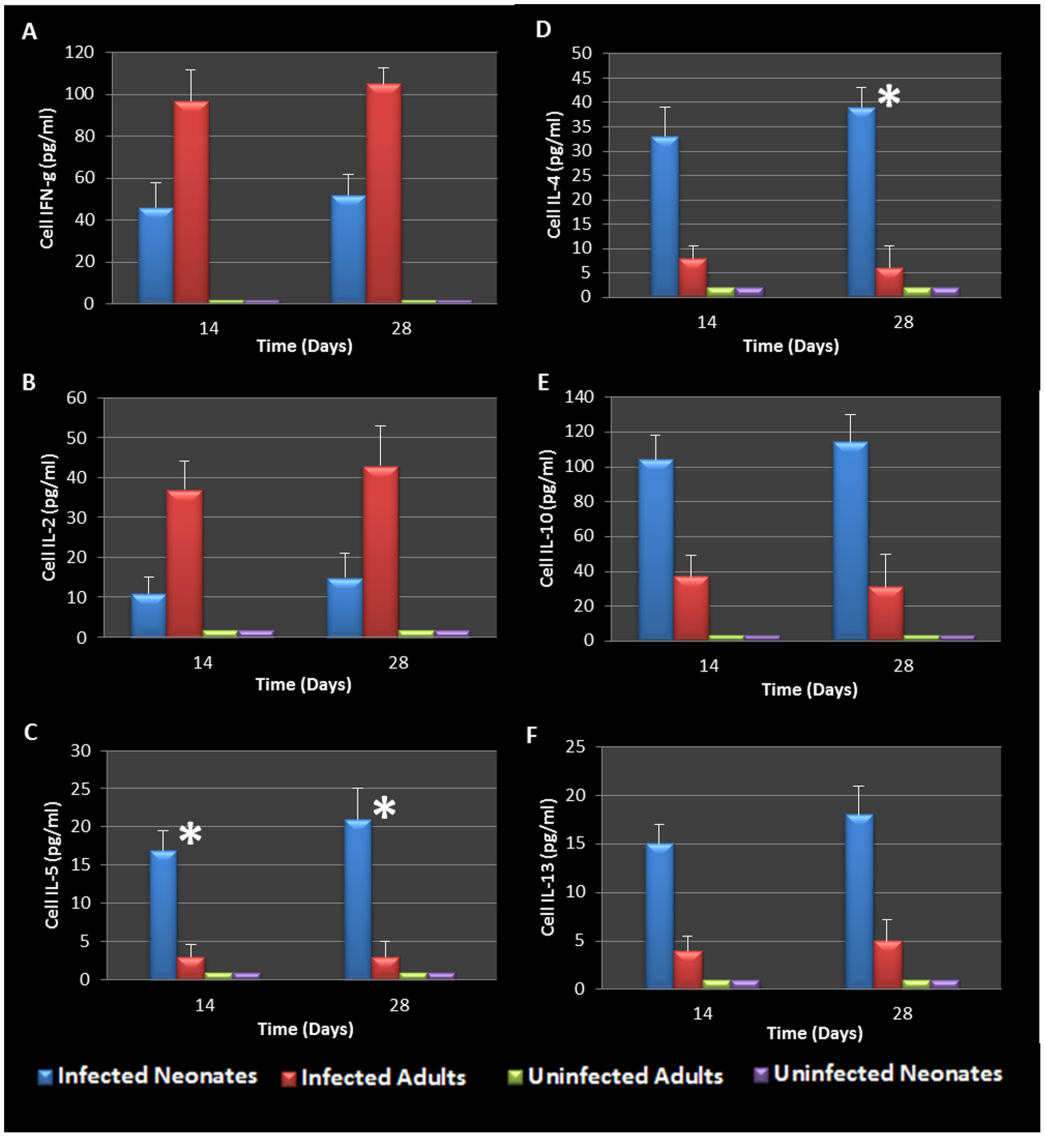
Mediastinal lymph node cell stimulation during chlamydial Infection. WBCs harvested from mediastinal lymph nodes were stimulated with heat killed chlamydial antigen and resulting cytokine secretions revealed that cells from infected adult mice secreted elevated levels of IFN-g and IL-2 when compared to infected neonates and controls (A, B). Cells harvested from infected neonatal mice secreted predominantly Th2 (IL-5, 4, 10, and 13) cytokines in response to chlamydial antigen compared to infected adults and control groups (C-F). WBCs from infected neonatal mice 14 days and 28 days post infection produced significantly elevated levels of IL-5 (P = 0.0353, 0.0456 respectively, C) and IL-4 (P = 0.0444, D) when compared to WBCs from infected adults. Cells were also stimulated with PBS and J774A.1 proteins as controls (data not shown).

BAL fluid was analyzed for the presence of Th1 and Th2 cytokines. The data confirmed that infected adults secreted significantly elevated amounts of Th1 cytokines (IFN-γ and IL-2) compared to uninfected animals (Figure 5A and B). However, infected neonates responded to *Chlamydia* airway infection with a robust Th2 cytokine response (IL-4, IL-10, IL-5, and IL-13) compared to their uninfected and adult infected counterparts (Figure 5C-F). Infected neonatal mice had significantly elevated IL-4 and IL-10 production compared to infected adults 28 days post infection (P = 0.0248, 0.0434 respectively). Mediastinal lymph node T cells from infected adult and neonatal mice were also isolated, cultured and stimulated with purified chlamydial elementary bodies to assess T- cell specific cytokine production. The cytokine profiles were similar to that seen in the BAL fluid, clearly highlighting the contrasting Th1 cytokine production seen in adult animals compared to the Th2 cytokine profile elicited by neonatal respiratory chlamydial infection (Figure 6). Specifically, neonatal mice had significantly more IL-5 and IL-4 cytokine production in response to chlamydial antigen (P = 0.0353, 0.0444 respectively), see Figure 6C and D. The magnitude of cytokine response in the tissue culture fluid from draining lymph node T cells was significantly greater than that seen in the BAL fluid.

### Cellular and Cytokine Response to Induction of Allergic Airway Disease in Neonatal and Adult Mice

**Figure 7:**
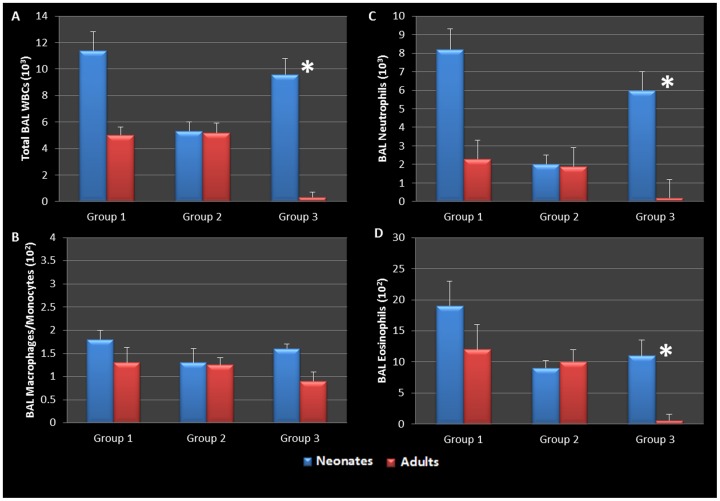
BAL cellularity during allergic airway disease. Infected neonatal groups with active infections had elevated levels of total WBCs, neutrophils, and eosinophils compared to all other groups (A, C, D). All groups which had allergic airway induced (AAD) with Ova had elevated levels of BAL eosinophils; however group 3 neonates who had active infections but did not have AAD induced had significantly elevated levels of BAL WBCs, neutrophils, and eosinophils when compared to their adult counter parts (P = 0.0001, 0.0003, and 0.0004 respectively); group 1 neonates who had active infections and AAD induced had enhanced levels of BAL WBCs, neutrophils, and eosinophils when compared to all other groups (A, C, D). There was no significant increase in BAL macrophage/monocytes in any group (B).

Having established that early life chlamydial infection induces different responses in neonatal compared to adult mice, we wanted to further determine if induction of allergic airway disease had different cytokine and cellular profiles as well. In this model, following respiratory infection with *Chlamydia*, mice were sensitized to Ova and some groups were challenged over several days while others remained unchallenged for comparison (Table 1). Our infected neonatal group (group 3 neonate) who still had active infections and no AAD induced, presented with significantly elevated numbers of total white blood cells, neutrophils and eosinophils, but low numbers of monocyte/macrophage cells when compared to their infected adult counterparts (group 3 adults) who did not have active infections (P = 0.0001, 0.0003, and 0.0004 respectively, Figure 7A, C, D). Indeed all groups which had allergic airway disease induced (see table 1) had airways characterized by elevated levels of BAL eosinophils; however, infected neonates who did not have AAD induced, had comparable levels of eosinophils in their BAL fluid compared to the AAD induced groups. The most significant elevation in eosinophils, was seen in neonatal animals that had active infections and AAD induced (Figure 7D). There was no significant increase in BAL macrophage/monocytes in any treatment group.

**Table 1:** Animal grouping and treatments.

Neonatal & Adult Groups	Description of Treatment
Group 1	Infected, Sensitized with Ova & Challenged
Group 2	Uninfected, Sensitized with Ova & Challenged
Group 3	Infected, No Sensitization & No Challenge

Sensitization and subsequent challenge with Ova albumin is required to induce allergic airway disease.

**Figure 8:**
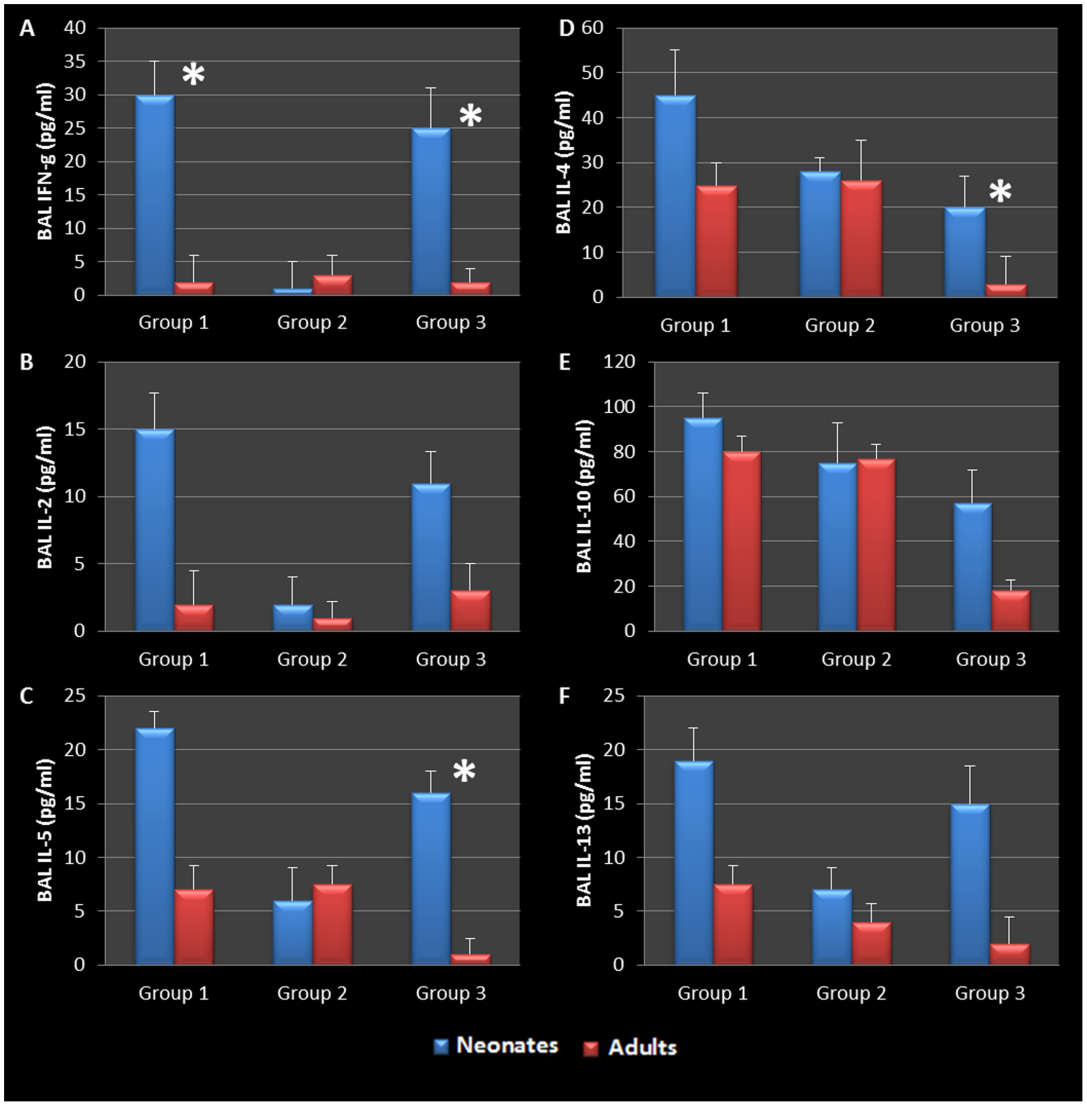
BAL cytokines during allergic airway disease. Infected adults who cleared infection, developed a mixed Th1/Th2 immune response during allergic airway induction (C-F). Group 1 and 3 infected neonates responded with a significant increase in IFN-g (P = 0.0049, 0.0092 respectively, A) and elevated levels of IL-2 when compared to corresponding adult groups (B). Th2 cytokines that were produced in all groups that had AAD induced (C-F). Infected neonates hyper reacted to ovalbumin compared to their adult and uninfected counterparts. Importantly to note, group 3 neonates who had active infections but no AAD produced significantly elevated amounts of IL-5 and IL-4 (P = 0.0152, 0.0362 respectively C, D) and elevated levels of IL-10, and 13 compared to all other groups (E,F). Collectively, the data therefore suggests that early life chlamydial infection induces an allergic airway response upon allergen challenge that is typical of asthma pathogenesis and supports a chronic infection model airway hyperresponsiveness.

BAL and mediastinal lymph node cytokine production in AAD induced animals was also assessed. Infected neonatal animals demonstrated a significant increase in the production of BAL Th2 cytokines (IL-4, -5, -10 and -13) in response to Ova challenge and sensitization, but also responded with the production Th1 cytokines (IFN-γ and IL-2) compared to their adult or uninfected counterparts (Figure 8). There was no statistical difference in the level of cytokines produced by infected neonatal mice that were not sensitized but later challenged with Ova (Group 3 neonates vs. Group 1 neonates in graphs, see table 1) when compared to the infected, sensitized and challenged group, suggesting that these animals might have become hyperresponsive as a result of early life chlamydial infection. In contrast, Group 3 neonates with active infections and no AAD, produced significantly elevated levels of IFN-g, IL-5, and IL-4 when compared to group 3 adults which had cleared infections and had no AAD (P = 0.0092, 0.0152, and 0.0362 respectively, Figure 8A, C, D). Though not significant, these neonates also presented with elevated IL-2, IL-10, and IL-13 when compared to adult mice, showing that chlamydial infection alone was enough to elicit a Th2-like response.

### Mediastinal Lymph Node T cell-specific Cytokine Response to *Chlamydia* and Ova Stimulation

**Figure 9:**
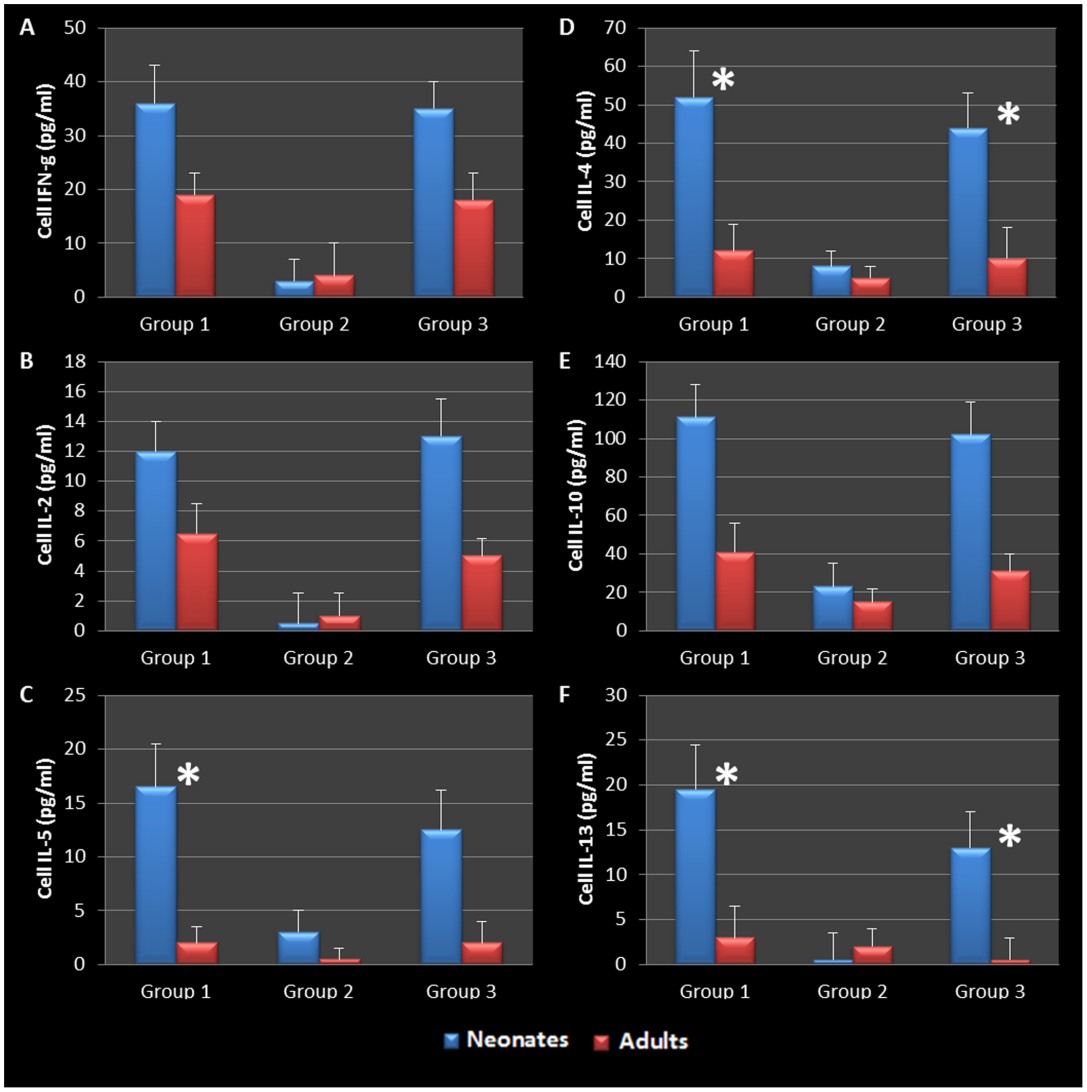
Mediastinal lymph node cell stimulation during allergic airway disease. WBCs harvested from mediastinal lymph nodes were stimulated as described previously described (Figure 5). Stimulation revealed that cells from infected neonatal mice with active infections (Groups 1 and 3) secreted elevated levels of IFN-g and IL-2 when compared to infected adults and controls (A, B). Cells harvested from infected neonatal mice secreted predominantly Th2 (IL-5, 4, 10, and 13) cytokines in response to chlamydial antigen compared to infected adults and control groups (C-F). Infected neonates with AAD (group 1) produced significantly more IL-5, IL-4, and IL-13 compared to corresponding adult groups (P = 0.0302, 0.0437, and 0.0362 respectively C, D, F). Infected neonates which did not have AAD were also able to produce significantly elevated levels of IL-4 and IL-13 compared to adults groups as well (P = 0.0456, 0.0075 respectively C, F). Cells were also stimulated with PBS and J774A.1 proteins as controls (data not shown).

**Figure 10:**
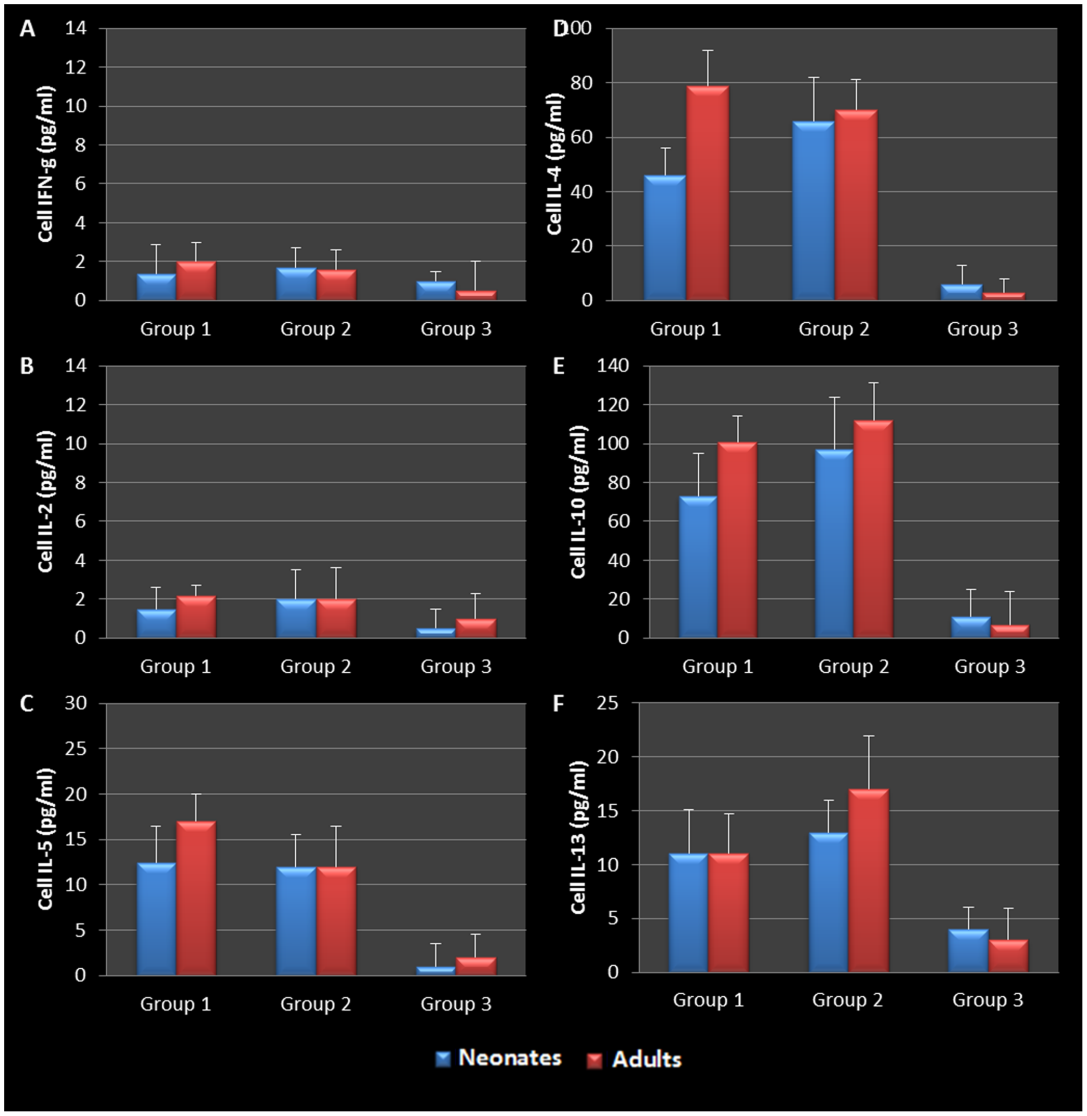
Mediastinal lymph node cell stimulation with OVA. WBCs harvested from mediastinal lymph nodes were stimulated as described previously described but ova albumin was used instead of heat killed chlamydial organisms. Stimulation revealed that cells from groups 1 and 2 that were sensitized and challenged with ova secreted predominantly Th2 (IL-5, 4, 10, and 13) cytokines in response to ova antigen compared to unsensitized/challenged control groups (C-F). Th1 cytokines (IFN-g, IL-2) were produced in negligible amounts in response to ova stimulation (A, B).

**Figure 11:**
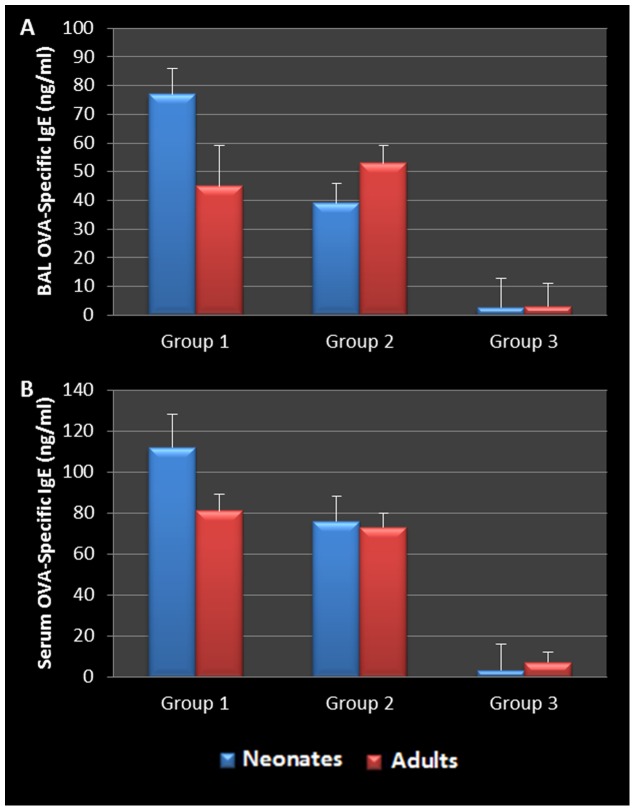
BAL Ova-Specific IgE During Allergic Airway Induction. Allergic airways were induced using ovalbumin. Groups which were both sensitized and challenged with ova had significantly elevated levels of ova-specific IgE antibodies present in BAL fluid (A) and serum (B), demonstrating successful allergic airway induction. Although not significant, infected neonatal group produced more ova-IgE than comparable adult group (Group 1).

**Figure 12:**
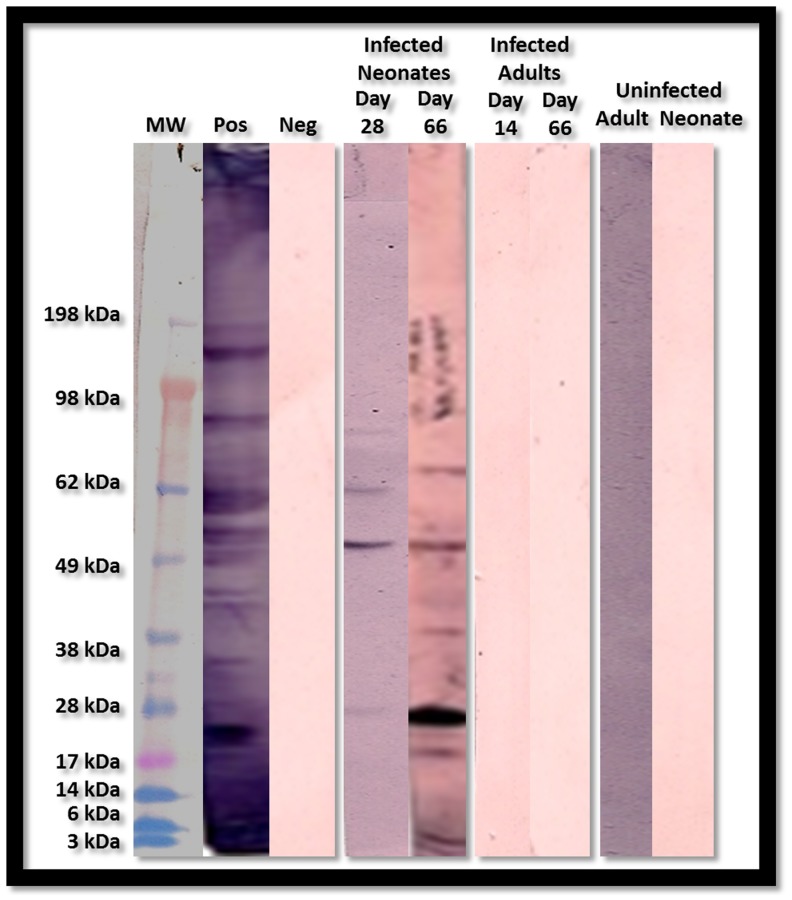
Detection of *Chlamydia* Specific IgE. Western Blot analysis revealed the presence of *Chlamydia*-specific IgE antibodies in the BAL fluid of infected neonatal groups only. Antibodies appeared as early as 28 days post infection and were still detectable 66 days post infection**.** *Chlamydia-*specific IgE antibodies appear to have been generated against a variety of chlamydial antigen representing a total of approximately 6 proteins. All proteins that induced IgE were either secreted or surface exposed.

To assess and confirm the specific immunological response to Ova stimulation, mediastinal lymph node T cells were cultured as previously described for assessment of *Chlamydia* and Ova antigen-specific cytokine. Mediastinal lymph node T cells from all *Chlamydia*-infected neonatal groups released elevated levels of IL-2, -10, IFN-γ (Figure 9A, B, E), and significantly elevated levels of IL-5, -4, -13 (P = 0.0302, 0.0437, and 0.0362 respectively, Figure 9C, D, F) in response to chlamydial antigen when compared to controls. By contrast, mediastinal lymph node T cells from similarly treated adult mice produced no significant levels of Th2 cytokines (IL-4, -5, -10 and -13), and much lower levels of IL-2 or IFN-γ when compared to similarly treated neonatal animals. Ova-specific T cells from Ova sensitized and challenged adult and neonatal animals produced significant levels of IL-4, -5, -10 and -13 in response to Ova stimulation in vitro. However, there was no production of the Th1- specific cytokines, IL-2 and IFN-γ (Figure 10 A -F). This was accompanied by a significant increase in the production of Ova-specific IgE antibodies in both the serum and BAL fluid of all sensitized and challenged groups (Figure 11). As controls, cells were also stimulated with cell lysate and sterile saline, resulting in no cytokine production. In addition, only infected neonatal mice produced *Chlamydia*-specific IgE antibodies when compared to all other groups. The level and antigen specificity of *Chlamydia*-specific IgE antibodies increased significantly over time as demonstrated by the band intensity and diversity on the Western blot image (Figure 12). Moreover, only approximately 6 chlamydial antigens appear to induce IgE production in this model, including the 40 kDa major outer membrane protein (Momp), chlamydial lipopolysaccharide (cLPS at 4–6 kDa), the *Chlamydia* lectin binding proteins (LBP, 18 and 27 kDa) and the cysteine rich protein A (CrpA, 15 kDa). Infected adult animals demonstrated no *Chlamydi*a-specific IgE in their sera, even at the time of sacrifice.

## Discussion

Asthma is a major public health problem and remains a significant burden on families and society in general. The most recent asthma surveillance data from the Centers for Disease Control and Prevention (CDC) report that 1 in 11 American children have the disease[19]. Asthma is the most common chronic disease of childhood and the numbers of young people and children with asthma continues to rise. While many cases of asthma are successfully managed and patients lead a relatively normal life, there is no cure for the disease and a growing number of severe, persistent asthmatics present with a phenotype that is refractive to oral or inhaled corticosteroid treatment. New reports confirm that patients with severe refractory asthma have the greatest unmet treatment needs to improve asthma control and reduce the risk of exacerbation [20]. In order to effectively treat, especially children with refractory asthma, it is necessary to better understand the etiology and or mechanisms involved in its development. Previous studies demonstrated that early-life respiratory viral infections, specifically with respiratory syncytial virus (RSV), increased the risk of subsequent development of childhood asthma [21]. In the model reported in that study, allergic airway inflammation, characterized by eosinophil recruitment and Th2 cytokine infiltration was prominent only in animals that had recovered from neonatal infection with pneumonia virus of mice and then later sensitized and chronically challenged with antigen [21].

In the current study we sought to better understand the underlying immunological mechanisms involved in the development of childhood asthma. We have demonstrated that while chlamydial organisms typically induce a Th1 type cytokine response with corresponding macrophage and neutrophil infiltration, neonatal respiratory infection led to production of Th2 dominant cytokines and infiltration of neutrophils and eosinophils in the airway. These results are similar to those previously reported by Horvat et al [15] using a similar Ova mouse model. In the current study however, we also demonstrated prolonged infection in neonatal animals that never cleared the bacteria from their lungs over the entire course of the study. While we utilized twice the inoculum (200 IFU) used by Horvet et al, this inoculum still represents less than what would typically be found in a single chlamydial inclusion, that can hold between 500 to 1000 EBs [22]. These findings suggest an interplay between the neonatal immune system and the chlamydial infection. The immaturity of the neonatal mouse immune system most likely altered the outcome of the infection leading to chronic infection and inflammation. At the same time, the infection most likely shaped the development of the immune system at least in the airway and could significantly impact its future responses. Clinically this could mean that in humans a lack of clearance of inflammatory infectious pathogens like C. *pneumoniae* following early-life lung infection could alter the airway structure and response. This could result in a more Th2 cytokine milieu, increasing the likelihood of developing allergic airway hyperresponsiveness to a variety allergens.

Mediastinal lymph node T cells and BAL fluid revealed a significant increase in Th1 and Th2 cytokines upon Ova sensitization and challenge in neonatal animals, but to a significantly less extent in adult animals who produced very little Th2 cytokines upon Ova sensitization and challenge. Indeed the levels of Th2 cytokines seen in neonatal animals that were infected and challenged without Ova sensitization were essentially similar to those of infected neonates who were both sensitized and later challenged with Ova. These data suggest that adult animals infected with *Chlamydia* in early life, display a hyperresponsive airway and a predisposition to allergic inflammatory diseases later in life. This hypothesis is supported by studies done by You, et al, demonstrating that severe RSV infection during neonatal development significantly altered lung structure and the pulmonary immune micro-environment, which potentially increased the risk of airway hyperresponsiveness later in life [23].

Neonatal mice never cleared the chlamydial organisms from their lungs and the bacteria disseminated to the liver and spleen and could also be isolated from the peripheral blood starting at 7 days post infection and at the time of euthanasia. This study also confirmed that mice infected as neonates but not adult animals, produced and released *Chlamydia*-specific IgE antibodies in both the serum and BAL fluid, while the adult mice did not. Since *Chlamydia*-specific IgE is being isolated from the blood as well as BAL fluid of these animals, the constant presence of the organism lends itself to an endless supply of these antigens which we presume will engage antigen-specific IgE on the surface of mast cells leading to the release of vasoactive amines, compounding airway hyperresponsiveness and increased lung pathology.

The data have important implications in asthma development and potential treatment or intervention for early-life respiratory infection. In a recent report by Seigle et al, the authors utilized a murine model of childhood asthma to demonstrate the utility of administration of anti-IL-4 or anti-IL-25 [24]. These antibodies prevented development of some key features of asthma, suggesting that suppression of a Th2 response during the neonatal period or later in childhood could be effective for preventing later chronic severe disease [24].

We therefore conclude that chlamydial airway infection early in development, can lead to significant alteration in lung structure and the immune architecture of the respiratory micro-environment. Early life *Chlamydia* infection was not cleared in this model, suggesting that this type of structural airway damage and immune modulation through continued secretion of Th2 cytokines continued unabated. Upon allergen exposure, the interaction between early-life chlamydial infection which results in chronic pulmonary inflammation and allergen sensitization exacerbates allergic airway disease through increased cytokine secretion and cellular infiltration. This combination has the potential to lead to more severe asthma that is refractory to inhaled steroids, since the root of the initial inflammation is a pathogen and could still be lurking in the lung tissue.
